# Towards Effective and Efficient Patient-Specific Quality Assurance for Spot Scanning Proton Therapy

**DOI:** 10.3390/cancers7020631

**Published:** 2015-04-13

**Authors:** X. Ronald. Zhu, Yupeng Li, Dennis Mackin, Heng Li, Falk Poenisch, Andrew K. Lee, Anita Mahajan, Steven J. Frank, Michael T. Gillin, Narayan Sahoo, Xiaodong Zhang

**Affiliations:** 1 Department of Radiation Physics, University of Texas M. D. Anderson Cancer Center, Houston, TX 77030, USA; E-Mails: Yupeng.Li@varian.com (Y.L.); dsmackin@mdanderson.org (D.M.); hengli@mdanderson.org (H.L.); fpoenisch@mdanderson.org (F.P.); mgillin@mdanderson.org (M.T.G.); nsahoo@mdanderson.org (N.S.); xizhang@mdanderson.org (X.Z.); 2 Department of Radiation Oncology, University of Texas M. D. Anderson Cancer Center, Houston, TX 77030, USA; E-Mails: Andrew.Lee@USOncology.com (A.K.L.); amahajan@mdanderson.org (A.M.); sjfrank@mdanderson.org (S.J.F.)

**Keywords:** spot scanning proton therapy, IMPT, SFO, SFIB, patient specific QA

## Abstract

An intensity-modulated proton therapy (IMPT) patient-specific quality assurance (PSQA) program based on measurement alone can be very time consuming due to the highly modulated dose distributions of IMPT fields. Incorporating independent dose calculation and treatment log file analysis could reduce the time required for measurements. In this article, we summarize our effort to develop an efficient and effective PSQA program that consists of three components: measurements, independent dose calculation, and analysis of patient-specific treatment delivery log files. Measurements included two-dimensional (2D) measurements using an ionization chamber array detector for each field delivered at the planned gantry angles with the electronic medical record (EMR) system in the QA mode and the accelerator control system (ACS) in the treatment mode, and additional measurements at depths for each field with the ACS in physics mode and without the EMR system. Dose distributions for each field in a water phantom were calculated independently using a recently developed in-house pencil beam algorithm and compared with those obtained using the treatment planning system (TPS). The treatment log file for each field was analyzed in terms of deviations in delivered spot positions from their planned positions using various statistical methods. Using this improved PSQA program, we were able to verify the integrity of the data transfer from the TPS to the EMR to the ACS, the dose calculation of the TPS, and the treatment delivery, including the dose delivered and spot positions. On the basis of this experience, we estimate that the in-room measurement time required for each complex IMPT case (e.g., a patient receiving bilateral IMPT for head and neck cancer) is less than 1 h using the improved PSQA program. Our experience demonstrates that it is possible to develop an efficient and effective PSQA program for IMPT with the equipment and resources available in the clinic.

## 1. Introduction

Proton therapy can be delivered using either scattering or scanning beam methods [1,2]. Currently, the most common method of delivering scanning beam proton therapy is spot scanning, in which a pencil beam (spot) is magnetically scanned laterally to the beam direction to create a large treatment field without introducing scattering elements into the beam path [3,4]. Monoenergetic pencil beams with different energies are used to create the desired dose distribution along the beam direction. The intensity of each pencil beam can be modulated to deliver intensity-modulated proton therapy (IMPT) [5,6]. To achieve the desired dose distribution in target volumes using IMPT, treatment planners use an inverse planning process to optimize the weights of individual spots.

There are two general approaches to optimizing a spot scanning proton therapy (SSPT) plan. The first approach is single-field optimization (SFO), in which the spot weights are optimized on a field-by-field basis; that is, each field is optimized individually to deliver a fraction of the prescribed doses to the entire target volume(s). SFO is most commonly used to produce a uniform dose over the entire target volume by each field, known as single-field uniform dose (SFUD); it is also used to create a simultaneously integrated boost by each field, called single-field integrated boost (SFIB) [7]. The second approach to optimize an SSPT plan is multiple-field optimization (MFO), in which the weights of all spots in all fields are optimized simultaneously to produce the desired dose distribution. This approach is also commonly known as IMPT.

One of the most important elements of a successful SSPT program is patient-specific quality assurance (PSQA). A PSQA program should be able to verify the dose calculated by the treatment planning system (TPS) and the correctness of treatment delivery. This verification is commonly achieved by comparing the calculated and measured dose distributions in a phantom. For SSPT, treatment delivery includes the variables specifying the spot position (lateral positions X and Y and longitudinal location Z determined by the energy of the beam for a given phantom), spot size, and amount of dose delivered by the spot (*i.e.*, the monitor unit (MU)).

We previously reported our PSQA experience for SFUD patients that was based on measurement only and derived from a PSQA program for intensity-modulated radiation therapy (IMRT) with photons [8]. The increased complexity of radiation treatment plans of IMPT has prompted us to measure doses at more depths to sample the complex dose distributions for each field. The additional measurements not only have significantly increased the measurement time, but also have made the data analysis very time consuming. This is a significant issue, as proton therapy centers in the United States operate for up to 16 h per weekday, which leaves little time for the treatment room to be available for PSQA activities.

The limited time available for PSQA merits the development of PSQA programs that can be executed efficiently. However, knowledge in this area is lacking, as few reports have comprehensively described PSQA programs for patients receiving particle therapy [6,8,9]. Most reports on PSQA programs only described specific aspects of such programs [10,11,12,13,14,15]. In this article, we review our efforts in developing an effective and efficient PSQA program for SSPT.

## 2. SSPT Beam Delivery System and TPS

**Figure 1:**
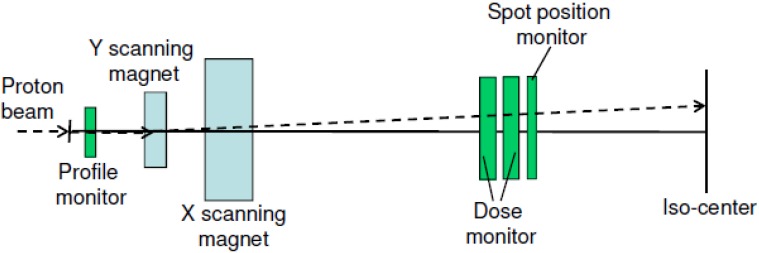
Schematic of the M. D. Anderson scanning nozzle. Major PSQA-related components are the profile monitor; Y and X scanning magnets; main dose and sub-dose monitors; and spot position monitor. The proton beam is indicated by the dashed line (from Figure 1 of [17] with permission).

The SSPT beam delivery system at the University of Texas M. D. Anderson Cancer Center consists of a synchrotron (PROBEAT Proton Beam Therapy System, Hitachi America, Ltd., Tarrytown, NY, USA) with 94 energies ranging from 72.5 MeV to 221.8 MeV; these energies correspond to proton ranges of 4.0–30.6 g/cm^2^ in water [4,16]. The maximum field size is 30 × 30 cm at isocenter. For patients with shallow target volumes (<4 cm^2^/g at the distal end of the target), a 6.7-cm range shifter made of acrylonitrile butadiene styrene resin is used to bring the Bragg peak as shallow as 0.3 cm^2^/g. A schematic of the M. D. Anderson scanning nozzle and its major PSQA-related components is shown in Figure 1.

We used the Eclipse TPS (versions 8.120 and 8.917, Varian Medical Systems, Palo Alto, CA, USA), which has a proton module for double scattering and SSPT delivery, in this report. The TPS uses a pencil beam algorithm. The commissioning of the TPS’s dose models in water for SSPT has been described previously [17].

As of April 2014, more than 1200 patients had been treated with SSPT at our proton therapy center. These patients’ disease sites included the prostate (64% of patients), central nervous system and pediatric (14%), head and neck (15%), and lung (6%). About 16% of these patients were treated with IMPT, 11% were treated with SFIB, and the remaining 73% were treated with SFUD.

## 3. Measurement-Based PSQA

### 3.1. Prostate Patients Treated with SFUD

While it was initially developed based on photon IMRT [8], our PSQA program for SSPT differs from that for photon IMRT because of the finite ranges of proton beams. If the fluence of a photon beam is known at one depth, its fluence at other depths can be determined, which suggests that measurements done at one depth can provide sufficient information to determine whether the field is delivered correctly. This cannot be said for a proton beam because protons with different energies stop at different depths. In addition, the phantom size matters for proton beams. It is nearly impossible to make composite dose measurements with all the beams applied to a phantom unless the phantom is approximately the same size as the patient and the detector can be freely moved to a proper position in the phantom. These two differences led us to develop a PSQA program for SSPT that is performed on a field-by-field basis.

**Figure 2:**
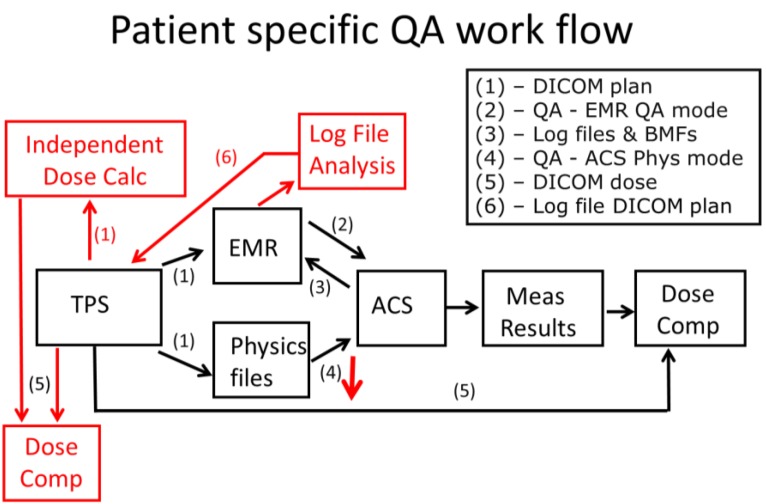
PSQA workflow. Black indicates the measurement-based workflow; red indicates the independent dose calculation, HPlusQA, and log file analysis.

**Figure 3:**
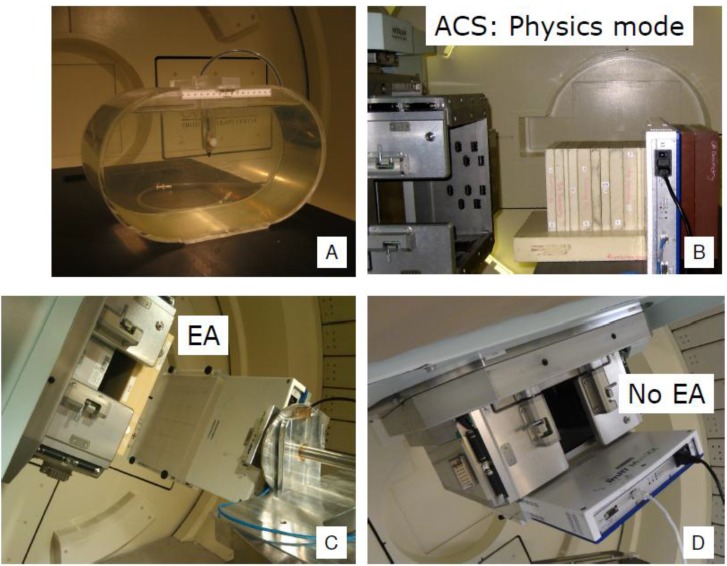
PSQA measurement apparatus. (**A**) A water phantom used for point dose measurement for prostate patients; (**B**) 2D-ICAD for measurements at different depths; and Couch mounted (**C**) and gantry mounted (**D**) 2D-ICAD for measurements of fields with a range shifter at the treatment gantry angle.

As shown in Figure 2, PSQA measurements consist of two components: [1] dose measurements using the treatment fields delivered through the electronic medical record (EMR) system (Mosaiq versions 1.5–2.4; Elekta Medical Systems, Sunnyvale, CA, USA) in the QA mode and through the accelerator control system (ACS) in the treatment mode; and [2] additional dose measurements of depth dose and two-dimensional (2D) dose distributions at different depths in the physics mode of the ACS. The most important element of the first component accomplishes the end-to-end test of data transfer integrity from the TPS to the ACS. The second component includes additional measurements at different depths in the physics mode to further validate the dose model. Phantoms used for the measurements are shown in Figure 3.

For the first 249 prostate cancer patients we treated with SFUD using this PSQA program [8], the difference between the measured and calculated point doses (mean±standard deviation) was 0.0% ± 0.7%. We also established a 90% passing rate using the 3%/3 mm γ-index criteria for 2D dose comparison. It should be pointed out that we use 2D γ-index in this article unless otherwise specifically stated. The 3% criterion is global with 10% as the cut-off of the low dose threshold. Using the QA measurements, we also confirmed some limitations of the dose model used for these patients, which led us to develop an improved dose model that uses a double Gaussian function for the fluence of individual spots [17].

### 3.2. Patients with Target Volumes Outside the Pelvis: SFO and MFO Plans

**Figure 4:**
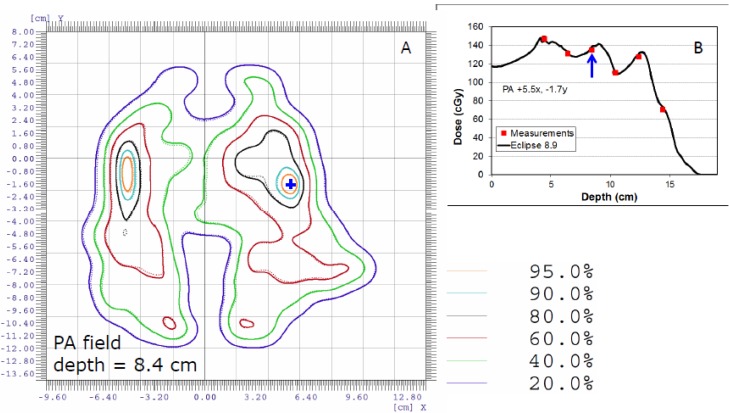
Comparison of 2D isodose (**A**) and depth doses (**B**) between measured and calculated doses along the off-axis point indicated by the blue cross in (**A**) for the PA field of a head and neck cancer patient. The percentage of voxels that passed the γ-index with a 2% dose/2-mm criteria was 99.9%. Measurements (indicated with red squares) were performed at five different depths.

After improving the dose model [17] and commissioning the range shifter for SSPT, we started to treat patients who had target volumes in the brain and head and neck. Initially, we simply extended our PSQA program for prostate cancer patients to other disease sites of patients with the following modifications: First, for treatment fields delivered through the EMR system, the point dose measurements obtained in the phantom used for prostate patients (Figure 3A) were replaced with 2D measurements using a 2D-ionization chamber array detector (2D-ICAD) (MatriXX^TM^, ScanditronixWellhofer, Schwarzenbruck, Germany) mounted either to a couch attachment or in the gantry for fields with a range shifter (Figure 3C) and fields without a range shifter (Figure 3D), respectively. Second, for complex dose distributions, more measurements were performed at different depths using the setup shown in Figure 3B. An example of a measured complex dose distribution compared with a TPS-calculated one for the posterior-anterior (PA) field of a patient with squamous cell carcinoma of the right base of the tongue is shown in Figure 4.

**Figure 5:**
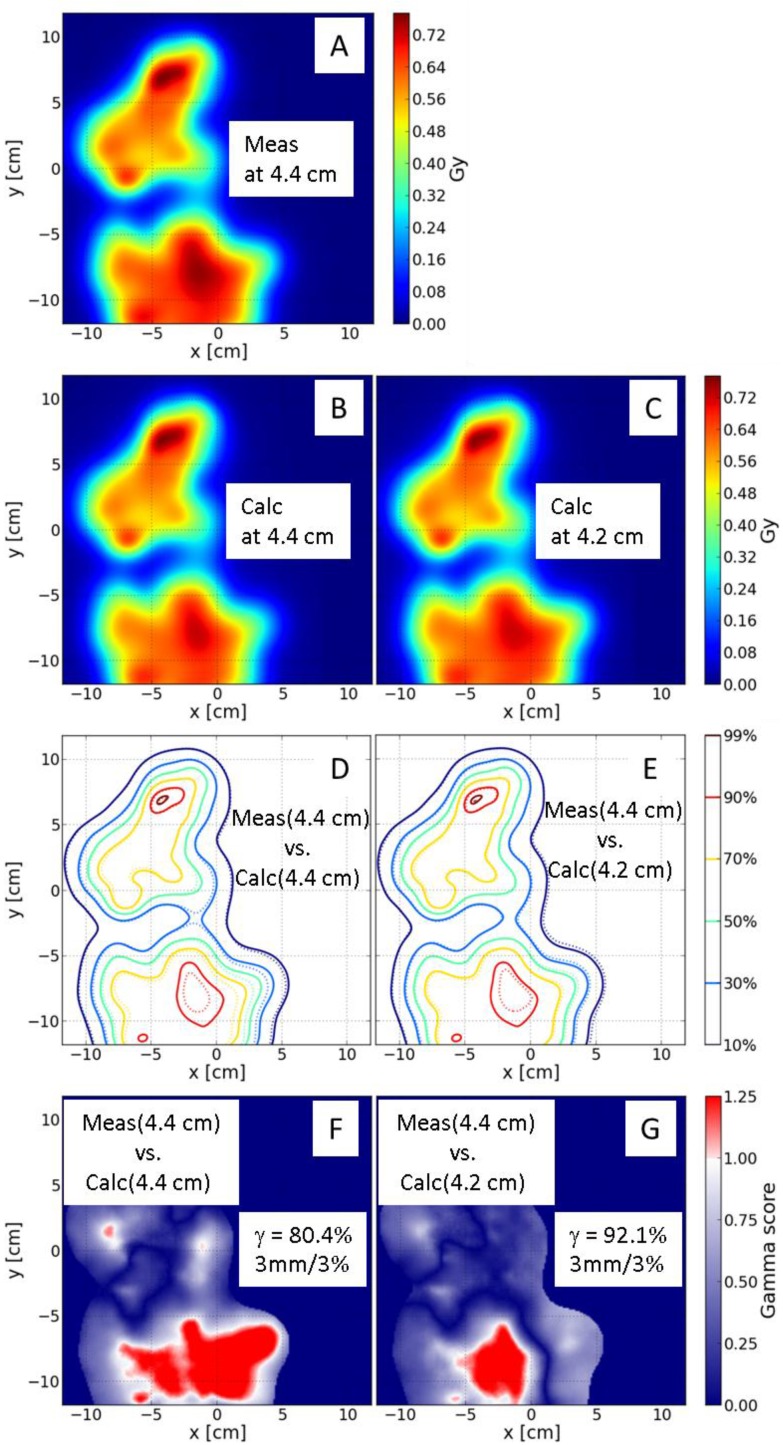
2.5D γ-index analysis for a left superior-inferior IMPT field of a head and neck patient. (**A**) Measured doses at the depth of 4.4 cm; Calculated doses at depths of 4.4 cm (**B**) and 4.2 cm (**C**); Comparison of measured with calculated isodose lines at the depth of 4.4 cm (**D**) and measured at the depth of 4.4 cm with calculated at the depth of 4.2 cm (**E**) (Solid lines are measured and dashed lines are calculated); γ-index distributions for the comparisons at the depth of 4.4 cm (**F**) and the measured at 4.4 cm *versus* calculated at 4.2 cm (**G**).

Using the improved dose model, we found that the γ-index using the 3%/3 mm criteria often yielded a passing rate of close to 100%, even in a plane with a complex dose distribution (Figure 4A). Although the minimum passing rate of 90% using the 3%/3 mm criteria remained the same, we also included 2%/2 mm criteria to compare the measured and calculated dose distributions. If the passing rate was less than 90% using the 2%/2 mm criteria, we reviewed the γ-index using the 3%/3 mm criteria. If the passing rate was less than 90% using the 3%/3 mm criteria, we compared the measured dose distributions with the calculated dose distributions in the planes up to 3 mm deeper and shallower than the nominal depth. This could be considered as a 2.5D γ-index analysis. Figure 5 is an example for a left superior-inferior IMPT field of a head and neck patient. The measured (Figure 5A) and calculated (Figure 5B) 2D dose distributions at the depth of 4.4 cm were first compared. The passing rate of γ-index using the 3%/3 mm criteria was only 80.4% (Figure 5D,F). This was because a part of the plane at the depth of 4.4 cm was located in the distal region with high dose gradient. The measured dose distribution at 4.4 cm was then compared to the calculated ones at several different depths. Shown in Figure 5C is the calculated dose at the depth of 4.2 cm and Figure 5E is a comparison isodose distribution between measured dose at 4.4 cm and calculated dose at 4.2 cm. The passing rate of the γ-index using the 3%/3 mm criteria increased to 92.1% (Figure 5G) for the comparison in Figure 5E.

Measuring 2D dose distributions at many depths is very time-consuming but necessary if the QA program is based on measurement only. Performing measurements at one or two depths for such a complex dose distribution does not reveal whether the doses at the other depths are within the tolerance. Even when measurements are taken at several depths (e.g., at five depths, as in Figure 4B), there is no guarantee the 3D dose distributions would be completely acceptable for treatment. However, the likelihood of delivering an incorrect dose decreases, obviously, with dose distributions at more depths being verified with measurements. Our goal was to develop an effective and efficient PSQA program using the currently available tools in the clinic by minimizing the number of measurements without sacrificing the confidence that verifying the dose distribution for each field in a phantom provides. A PSQA program that includes an independent dose calculation and delivery log file analysis would help us achieve our goal.

## 4. A Combined Approach for PSQA

We improved our measurement-based PSQA workflow (Figure 2; black parts) by adding an independent dose calculation and delivery log file analysis (Figure 2; red parts). The dose calculation of the clinical TPS is verified with the independent dose calculation and planar dose measurements at selected depths. The treatment delivery is verified by dose measurement and delivery log file analysis. At first glance, the additional steps make the PSQA workflow more complex. However, the revised PSQA program has the key advantage of reducing or possibly eliminating the measurements in the physics mode (Figure 2; step 4). Recently, we further improved the efficiency of the program by automating the comparison of measured and calculated dose distributions and the generation of a draft PSQA report [15].

### 4.1. Independent Dose Calculation

Similar to the dose algorithm used by our clinical TPS, the independent dose calculation was a pencil beam dose algorithm using integral depth doses and lateral dose profiles [15]. The dose calculation is independent because different methods used to model the integral depth doses and lateral dose profiles. The integral depth dose curves were determined by simultaneously fitting seven measured Bragg curves with different energies to the modified Bortfeld [18] analytical Bragg curve formula [19]. The lateral dose profiles were modeled using Moliere scattering with two Gaussian functions and large-angle scattering with a modified Cauchy-Lorentz distribution [20]. The details of the implementation of independent dose calculation in the PSQA program, called HPlusQA, have recently been described by Mackin *et al.* [15]. The independent dose calculation was validated with more than 100 measured planar dose distributions. HPlusQA was found to be reasonably effective (79% ± 10%) in determining whether the comparison between the measured and TPS-calculated dose planes exceeded the acceptable tolerance levels. As an independent dose calculation method, HPlusQA can reduce the need for PSQA measurements by 64% [15].

**Figure 6:**
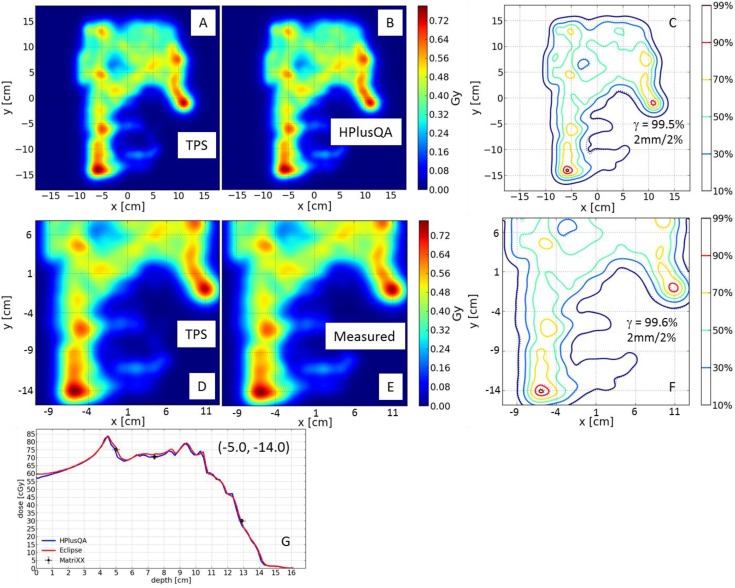
HPlusQA output of one of the fields for a patient with mesothelioma of the right hemithorax. TPS (**A**) and HPlusQA (**B**) calculated dose distributions in color wash. (**C**) Isodose comparison of (**A**) and (**B**); the percentage of voxels that passed the γ-index with a 2% dose/2-mm criteria was 99.5%; solid lines indicate HPlusQA, and dashed lines indicate TPS. TPS-calculated (**D**) and 2D-ICAD-measured (**E**) dose distributions in color wash; the size of the field of view was scaled to match the measured dose distribution. (**F**) Isodose comparison of (**D**) and (**E**); the percentage of voxels that passed the γ-index with a 2% dose/2-mm criteria was 99.6% ; solid lines indicate 2D-ICAD-measured dose distributions, and dashed lines indicate-TPS calculated dose distributions. (**G**) Depth-dose comparison of TPS-calculated dose distributions (red), HPlusQA-calculated dose distributions (blue), and 2D-ICAD-measured doses for coordinates (−5 cm, −14 cm) away from central axis (0, 0). Measurements (indicated with black points) were performed at three different depths.

It was proposed that the HPlusQA-calculated dose distribution could be compared with the TPS-calculated dose distribution as soon as the physician approved the treatment plan. The plan could be rejected if significant discrepancies existed between the TPS- and HPlusQA-calculated dose distributions, assuming there was higher chance of failing the QA process. The TPS- and HPlusQA-calculated dose distributions and the measured dose distributions were compared automatically using a few user-entered parameters, such as measurement depth and machine output. An example of such a comparison for one of the fields for a patient with a mesothelioma of the right hemithorax is shown in Figure 6, which again demonstrates the complex nature of dose distributions in IMPT fields.

In a follow up paper, Mackin *et al.* [21] retrospectively analyzed quality assurance results of 309 SSPT plans in the HPlusQA system. The overall γ-index passing rate was 96.2% with 3%/3 mm criteria, and reduced to 85.3% with 2%/2 mm criteria. The authors also reported γ-index passing rate was disease site (95% *vs.* 100% head neck *vs.* prostate) and range shifter (94.8% *vs.* 99.0% with *vs.* without range shifter) dependent but independent of the optimization methods SFO *vs.* MFO. The steep dose gradients perpendicular to the beam measurement plane were the major source of low γ-index scores. The authors also confirmed that γ-index passing rate 90% with 3%/3 mm criteria was a reasonable clinical action level for 2-dimensional comparisons of dose planes even for more complex dose distributions encountered in SFIB and IMPT.

### 4.2. Analysis of Patient-Specific Treatment Delivery Log Files

**Figure 7:**
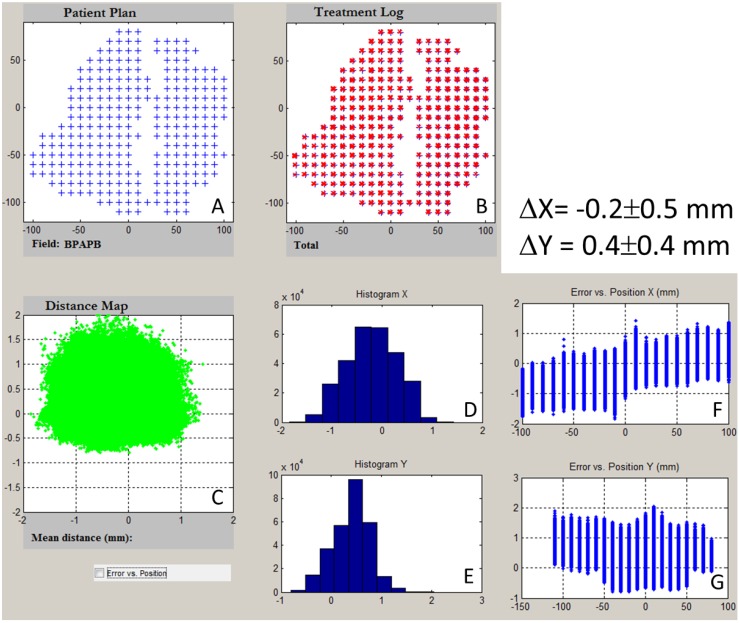
Log file of the posterior-anterior field of a head and neck cancer patient. (**A**) Planned spot positions for all energy layers; (**B**) Recorded spot positions for all energy layers in 33 fractions; (**C**) Deviation map from the planned spot positions; Histogram of deviation as a function of X (**D**) and Y (**E**) coordinates; and deviation as a function of X (**F**) and Y (**G**) coordinates.

We recently reported that treatment delivery log files containing delivered spot positions and MUs could be used as part of a PSQA program [14]. We found that spot positions in a given treatment session were reproducible to within 0.2 mm; the measured spots on film agreed with the planned position to within 1 mm and with the recorded positions to within 0.5 mm. The recorded patient spot positions and MU values were also used in place of the planned spot positions and MU values, and the TPS was used to calculate the doses delivered to patients to within 2%. (An example of a spot-log analysis for a patient with squamous cell carcinoma of the left tonsil is shown in Figure 7). Since the publication of that work, we have established more comprehensive spot position patterns as part of a periodic QA for comparing the measured spot positions and spot log results. These patterns include squares and diagonals as well as perturbed squares and diagonals with perturbations of 1, 2, and 3 mm for some spots.

## 5. Use of the Treatment Room for PSQA

The lack of treatment room availability for PSQA measurements can cause a bottleneck in starting new patients’ treatment. We reviewed the amount of time the treatment room was used for PSQA for 10 oropharynx patients with bilateral target volumes, including supra-clavicle nodes, treated with IMPT at three different dose levels (70, 63, and 57 Gy (RBE)) to the CTV1, CTV2 and CTV3, respectively). All patients were treated with a technique involving three fields: the right superior-inferior anterior oblique, the left superior-inferior anterior oblique, and the posterior to anterior fields. The time (mean ± 1 standard deviation) required for each measurement, which included the beam-on time, the time spent waiting for the beam to become available, and the time spent changing certain machine settings and/or QA setup as necessary, was 6.7 ± 1.7 min. The time for each measurement also depended on the performance of the machine (the number of times beam delivery was paused or aborted during beam delivery). The time required to set up and calibrate the 2D-ICAD was 33 ± 3 min. This time did not include the time required to warm up the detector, which was done before the treatment room became available.

As mentioned above, to obtain the required magnetic bending strength for each energy type, our SSPT delivery system requires each treatment field to be delivered through the EMR system in the QA mode and through the ACS in the treatment mode at least once. Given these circumstances, the minimum time required for use of the treatment room for PSQA measurement for one oropharynx patient is about 50 min. This time would be reduced to about 30 min per patient if three patients were measured during the same session because the time required to set up and calibrate the detector is reduced on a per-patient basis. Because we perform two additional measurements in the physics mode for each field, the treatment room time required for PSQA for one oropharynx patient would be about 100 min. This time would be reduced to approximately 70 min per patient if three patients were measured per session. Although the time is still long to use the treatment room, it represents significant progress compared to the PSQA time required for the first group of 10 oropharynx IMPT patients we treated (approximately 200 min for one patient only and about 170 min per patient if three patients were measured per session), for whom we took up to seven additional measurements for each field in the physics mode. Eliminating the measurements in the physics mode would reduce the treatment room time required for each patient to approximately 30–50 min depending on the number of patients measured per session. These results are specific to oropharynx patients with bilateral target volumes treated with a three-field IMPT technique. For other types of patients, these numbers may vary significantly depending on the complexity of the target volumes and the numbers of fields used.

## 6. Discussion

We have demonstrated that it is possible to develop an effective and efficient PSQA program for SSPT patients using the resources available to us in the clinic at M. D. Anderson.

The purpose of PSQA program was to ensure the planned dose was delivered to patient accurately. The ideal PSQA program for SSPT should be based on reliable and accurate 3D dosimeters to perform end-to-end test measurements for each field through the EMR in QA mode and ACS in clinical mode. However, 3D dosimeters are not mature enough to be used for routine QA, especially for proton beam therapy, despite active research in several different fronts, including liquid [22,23] and solid [24] scintillation dosimeters, solid dosimeters such as the PRESAGE dosimeter (a clear polyurethane matrix doped with radiochromic leuco dyes) [25,26], and polymer gel dosimeters [27,28]. Liquid scintillation dosimeters were recently reported to exhibit a nonlinear response around Bragg peaks, where the linear energy transfer (LET) values are high; despite the use of correction factors derived from a quenching model and LET data, the calculated and measured Bragg peak heights could still differ up to 10% [23]. Similar to liquid scintillation dosimeters, the PRESAGE dosimeter also shows significant underdose around the Bragg peak owing to LET effects [25,26]. The dose response at the distal end of polymer gel dosimeters may depend on variations in the gel composition [27]. On the other hand, 2D dosimeters such as 2D-ICAD have been commercially available for the last decade and have become a workhorse for PSQA [8,10].

For dose distributions measured with 2D dosimeters, 3D γ-analysis may be necessary, especially for the measurement plane near the steep dose gradient region such as the distal region in the example shown in Figure 5. However, the most current commercial software packages do not provide 3D γ-analysis tool probably due to fact that this type of software package is commonly used for photon IMRT. For IMPT applications, the need for 3D γ-analysis is very obvious.

The literature on comprehensive PSQA programs for patients receiving particle therapy is limited [6,8,9]. Some publications have reported only specific aspects of PSQA [10,11,12,13,14,15]. For example, some works focused on either dosimetric detector development or evaluation: Karger *et al.* [13] developed a detector based on a block of ionization chambers for 3D dosimetric verification of treatment plans in intensity-modulated heavy ion therapy, and Arjomandy *et al.* [10] reported the use of a 2D-ICAD for verification of patient-specific dose distributions in proton therapy. Other studies investigated the validation of the TPS for particle therapy. Iqbal *et al.* [11] reported QA evaluation of SSPT with an anthropomorphic prostate phantom, and Jäkel *et al.* [12] published QA for a TPS for scanned ion beam therapy. Lomax *et al.* [6] summarized their experience with an SSPT PSQA program, which included treatment field-specific dose verifications, for 166 patients. The authors measured dose profiles using an orthogonal ionization chamber array, typically at two different depths in water for each field. On average, the measured doses were about 1% less than calculated doses. The measured proton beam range was 1.5 mm more than the calculated one was, indicating a possible systematic error. Given the system’s reliability, the authors proposed reducing the number of measurements and quasi-randomly select fields for detailed measurements with an ionization chamber and a scintillating screen/charge-coupled device camera. Furukawa *et al.* [9] reported their experience with a PSQA program for scanning beam carbon ion therapy for 122 patients, which included more than 1400 measurements for 470 fields. They used a different 2D-ICAD (Octavius Detector 729 XDR, PTW, Freiburg, Germany) to perform measurements at several depths and evaluated their results using γ-index analysis with 3%/3 mm criteria and a pass rate of 90%. They developed a multiwire proportional chamber capable of recording the delivered 2D fluence images in a layer-by-layer manner during the dosimetric measurement. These 2D fluence images were used as the references in daily treatments for constancy check.

Independent dose calculations have been used as part of PSQA for IMRT with photons since the early days of IMRT [29,30,31,32,33,34,35,36,37,38]. These independent calculations provide additional confidence in dose calculations for all IMRT delivery techniques, including tomotherapy, and volumetric modulated arc therapy [38]. One recent study reported that an efficient and reliable 3D dose quality assurance program for IMRT could be achieved by combining independent dose calculation with measurements [39]. Literature on the independent dose calculation as part of PSQA for SSPT is very limited [15]. If the dose accuracies of the TPS were completely validated during the system’s commissioning, the PSQA measurements should only focus on the treatment delivery aspect. However, exhaustively validating the dose calculation of the TPS for various clinical scenarios during commissioning is nearly impossible; therefore, the validation of dose distribution of each treatment plan and fields is highly desirable. If the dose distribution could be independently verified with a high degree of confidence (e.g., by using experimentally verified Monte Carlo simulation), one could focus on the PSQA measurements of delivery of each energy layer and perform measurements in air or at a very shallow depth using a 2D detector such as a scintillation screen. The limitation of this approach is that it does not directly confirm the spot positions in the beam direction or the energy of the pencil beam for each patient. However, this limitation could be mitigated by implementing a more stringent periodic QA program such as monthly or weekly verification of beam energy.

The use of treatment delivery log files as part of PSQA for photon IMRT has been studied extensively [38,40,41,42,43,44,45,46,47]. In contrast, few studies have investigated using log files for SSPT PSQA [9,14]. The major criticism of using log files for PSQA is that the content of the log files is generated by the treatment delivery machine and thus not independent. If the device detecting possible delivery errors were to malfunction, it would either provide false positive or false negative results. Therefore, we implemented “QA of the QA” periodic QA in which dose measurements are used to verify the information contained in the log file. Because it was verified by independent dose measurements using an external dosimeter prior to the patient’s treatment, the log file generated during the QA measurements delivered through the EMR system in the QA mode and through the ACS in the treatment mode can serve as the reference for the entire course of treatment delivery. Furukawa *et al.* reported using a similar approach for carbon ion beam therapy, in which a multiwire proportional chamber was used to record the fluence on a layer-by-layer basis [9].

## 7. Summary

Our PSQA program for SSPT combining measurements [8], independent dose calculations [15], and machine delivery log file analysis [14] to detect errors associated with dose calculation and variables for spot delivery, including the position of the spots, energy, and dose and MU, is more effectively and efficiently than an approach based on measurement alone. Our ultimate goal is to perform the end-to-end QA test with a measurement for each field with the EMR system in QA mode and the ACS in clinical mode. Other aspects of verification can be performed through independent calculation, log file analysis, and machine QA. To achieve such verification, we must improve the independent dose calculation algorithms, including the use of Monte Carlo simulation dose calculation, and enhance the periodic machine QA to include certain aspects of PSQA, such as verification of pencil beam energies.
